# STING inhibition suppresses microglia-mediated synapses engulfment and alleviates motor functional deficits after stroke

**DOI:** 10.1186/s12974-024-03086-8

**Published:** 2024-04-08

**Authors:** Chaoran Wu, Shiwen Zhang, Hao Sun, Ao Li, Fengsheng Hou, Long Qi, Hong Liao

**Affiliations:** New Drug Screening Center, State Key Laboratory of Natural Medicines, China Pharmaceutical University, 24 Tongjiaxiang, Nanjing, 210009 China

**Keywords:** Ischemic stroke, Microglia, Synapse phagocytosis, STING, STAT1

## Abstract

Ischemic stroke is the leading cause of adult disability. Ischemia leads to progressive neuronal death and synapse loss. The engulfment of stressed synapses by microglia further contributes to the disruption of the surviving neuronal network and related brain function. Unfortunately, there is currently no effective target for suppressing the microglia-mediated synapse engulfment. Stimulator of interferon genes (STING) is an important participant in innate immune response. In the brain, microglia are the primary cell type that mediate immune response after brain insult. The intimate relationship between STING and microglia-mediated neuroinflammation has been gradually established. However, whether STING affects other functions of microglia remains elusive. In this study, we found that STING regulated microglial phagocytosis of synapses after photothrombotic stroke. The treatment of STING inhibitor H151 significantly improved the behavioral performance of injured mice in grid-walking test, cylinder test, and adhesive removal test after stroke. Moreover, the puncta number of engulfed SYP or PSD95 in microglia was reduced after consecutive H151 administration. Further analysis showed that the mRNA levels of several complement components and phagocytotic receptors were decreased after STING inhibition. Transcriptional factor STAT1 is known for regulating most of the decreased molecules. After STING inhibition, the nucleus translocation of phosphorylated STAT1 was also suppressed in microglia. Our data uncovered the novel regulatory effects of STING in microglial phagocytosis after stroke, and further emphasized STING as a potential drug-able target for post-stroke functional recovery.

**Supplementary Information:**

The online version contains supplementary material available at 10.1186/s12974-024-03086-8.

## Introduction

Stroke is characterized by a high disability rate, mortality rate, and recurrence rate, making it the second-leading cause of death and the third-leading cause of disability in adults worldwide [1]. The ischemia leads to progressive neural disconnection and network dysfunction, which ultimately results in the impairment of motor or sensory function [2]. Strategies to rescue the damaged neuronal network seem promising [3], however, the cellular and molecular mechanisms underlying the ischemia-induced neural disconnection are not fully explained and the effective drug-able targets remain to be identified.

After the onset of ischemia, the delivery of oxygen and nutrients is blocked and leads to massive neuronal death [4]. Accompanied by neuronal apoptosis, there is a rapid loss of dendritic spines around the infarcted region, indicating local neural disconnection [2, 3]. Progressively, the apoptotic events are reduced over time, but the synaptic density remains decreased [5]. It has recently been found that glial cells phagocytose synapses during the subacute phase of stroke and contribute to the ongoing damage of network connectivity [5, 6]. Conditional knockout of MEGF10 or MERTK in microglia could rescue the reduction of synapses and promote the recovery of motor function after MCAO injury [6]. The microglia-mediated synapse engulfment has also been shown to be complement-dependent, and complement inhibition could suppress synaptic uptake and prevent cognitive decline after reperfusion [5]. The increased synapse elimination by microglia has also been reported in various brain diseases, such as Alzheimer’s disease [7–9], schizophrenia [10], neuropsychiatric lupus [11], depression [12], and sepsis-associated encephalopathy [13], and is intimately involved in disease progression. These studies showed that microglia-mediated synapse engulfment plays a central role in injury-induced synapse loss and neuronal network dysfunction. Therefore, modulating microglia phagocytosis could be an effective strategy for neural circuit preservation and brain function recovery after stroke. However, there is still a lack of comprehensive understanding of synapse elimination by microglia and an effective therapeutic target.

Stimulator of interferon genes (STING) is a key participant in innate immune response. Upon injury or pathologic stimulus, nucleus or mitochondria DNA starts to accumulate and is sensed by cyclic GMP–AMP synthase (cGAS) or other potentially putative DNA sensors [14, 15]. The signal is then transduced to the ER-localized adaptor protein STING, which triggers its dimerization and translocation to the Golgi apparatus. STING then recruits and activates TANK-binding kinase 1 (TBK1) and interferon regulatory factor 3 (IRF3) to induce type I IFNs [16]. Microglia are the primary immune cells in the brain [17, 18]. Previous studies have mainly focused on the intimate relationship between STING and microglia-mediated neuroinflammation [19–23] or cell death [19, 23, 24]. For example, the released mtDNA induced by ischemic stroke has been proven to contribute to microglia polarization, and the usage of STING inhibitor C176 could promote microglia phenotype transition from pro-inflammatory M1 phenotype to anti-inflammatory M2 phenotype, which further contributes to infarction volume reduction and motor and cognitive function recovery [19, 22]. Moreover, STING knockout could also inhibit NLRP3-mediated microglial pyroptosis and ameliorate brain infarction and motor functional deficits in MCAO model [24]. Similarly, in other kinds of central nervous system diseases, such as Alzheimer’s disease [25, 26], Parkinson’s disease [27, 28], multiple sclerosis [29], neuropathic pain [30], ageing-related neurodegeneration [31], subarachnoid hemorrhage [32], and traumatic brain injury [33, 34], the regulatory role of STING in microglia-released inflammatory mediators has been thoroughly discussed. However, whether STING also regulates other important functions of microglia, like synapse pruning, has drawn little attention so far.

In this study, we investigated the potential relationship between STING and microglia-mediated synapse engulfment after stroke. We found that STING inhibitor H151 could significantly decrease phagocytosis-related molecules and suppress microglia eliminating synapses around the infarcted cortex, which promoted the preservation of ischemia-affected synapses and enhanced the recovery of stroke-induced motor dysfunction. Therefore, our study supported STING as a promising target for post-stroke recovery and proposed that STING could be an important regulator for synaptic phagocytosis by microglia under certain pathological conditions.

## Results

### Microglia in the peri-infarcted cortex upregulated STING after ischemic stroke

**Fig. 1:**
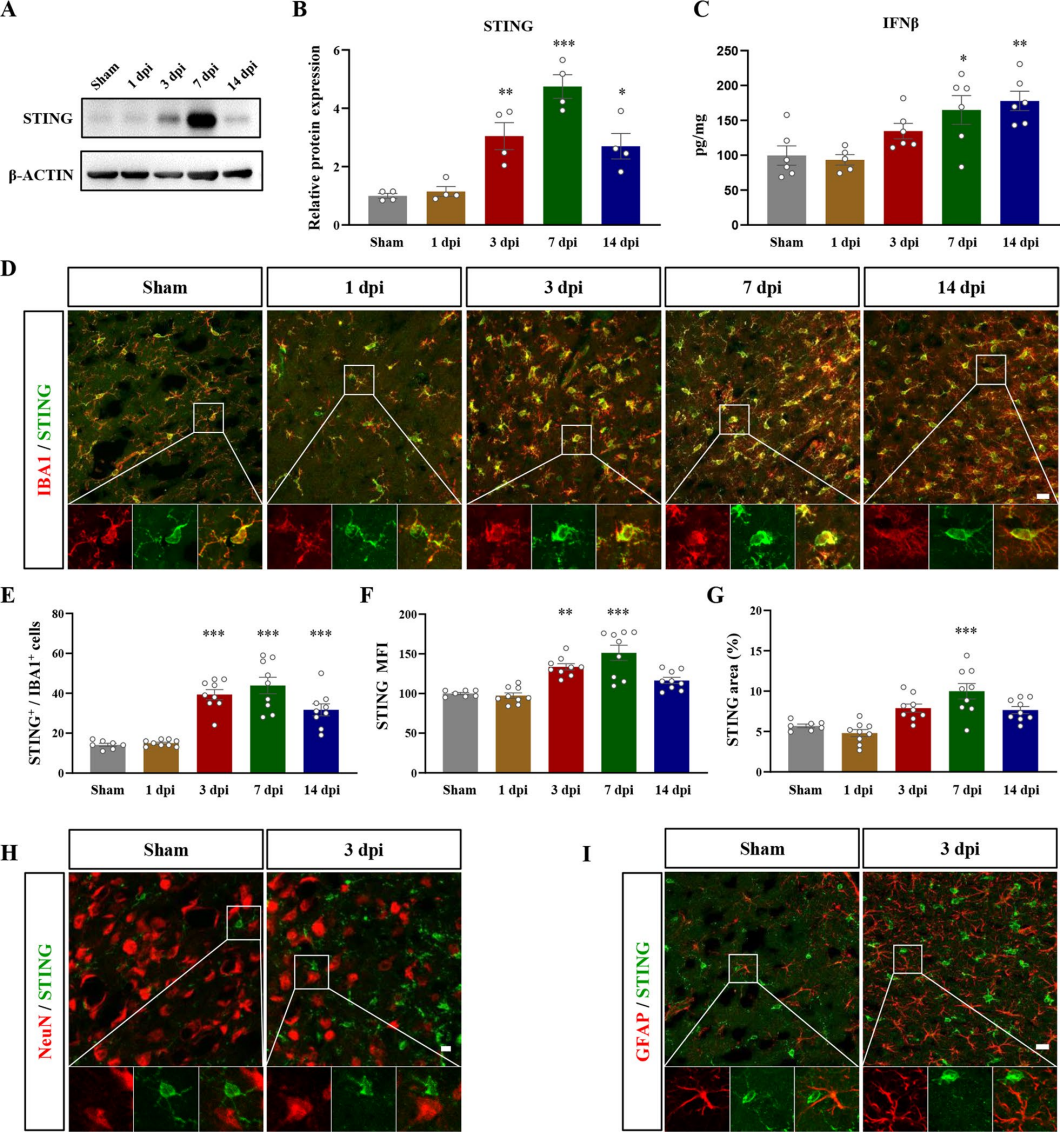
After stroke, STING was primarily expressed and upregulated in microglia. **A** Representative bands of STING and β-ACTIN using Western Blotting experiments. **B** The quantification of STING expression levels with β-ACTIN as the internal reference. The expression levels were relative to sham-treated group. n = 4 mice per condition. **C** The protein levels of IFNβ detected by ELISA. n = 5–6 mice. Each dot represented an individual mouse. **D** Representative immunofluorescent micrographs of STING and IBA1 at different time points after photothrombotic stroke. **E** The quantification of STING-positive microglia. Microglia were labeled by IBA1. **F** The mean fluorescence intensity (MFI) of STING relative to sham-treated group. **G** The quantification of STING-positive area. n = 7–9 field of view (FOV) from 3 mice per condition. Each dot represented a FOV. **H** and **I** Representative immunofluorescent micrographs of STING and neuronal marker NeuN (**H**) and astrocytic marker GFAP (**I**). Boxed regions were enlarged for analyzing colocalization. Scale bar = 20 μm (**D** and **I**). Scale bar = 10 μm (**H**). Data were presented as mean ± SEM. *P < 0.05, **P < 0.01, ***P < 0.001

We first examined the expression pattern of STING at different time points after photothrombotic stroke. At 3, 7, and 14 days post injury (dpi), the protein expression levels of STING were significantly upregulated relative to sham-treated group, peaking at 7 dpi (F(4, 15) = 19.58, *P* < 0.0001, Fig. 1A, B). We also detected an increment in cGAS expression levels at 3 and 7 days after stroke (F(4, 15) = 7.626, *P* = 0.0015, Additional file 1: Fig. S1A, B). IFNβ is the primary downstream effector of STING signaling [35], therefore, we detected the expression levels of IFNβ by ELISA around the infarcted cortex and found that IFNβ was also increased at 7 and 14 dpi (F(4, 24) = 6.729, *P* = 0.4813, Fig. 1C). Next, we investigated the cellular distribution of STING by immunofluorescence staining after stroke. We found obvious colocalization of STING and microglia marker IBA1 both in sham-treated group and stroke-injured group (Fig. 1D), while little or no colocalization was detected between STING and NeuN (Fig. 1H) or GFAP (Fig. 1I), indicating that STING was primarily expressed by microglia under both physiological and stroke-induced pathological conditions. Moreover, not all microglia expressed STING. From 3 days after stroke, the percentage of STING-positive microglia was dramatically increased (F(4, 38) = 25.42, *P* < 0.0001, Fig. 1E). The intensity of STING and the percentage of STING-positive area were also upregulated at 7 dpi (F(4, 38) = 17.38, *P* = 0.0076, Fig. 1F; F(4, 38) = 11.91, *P* < 0.0001, Fig. 1G), which was compatible with Western Blotting experiments (Fig. 1A, B). Together, these results showed that microglia was the primary cell type that expressed STING, and photothrombotic stroke could result in elevated expression levels of STING in microglia during the subacute phase of stroke.

### STING inhibitor H151 effectively suppressed STING activation and promoted motor function recovery after stroke

**Fig. 2:**
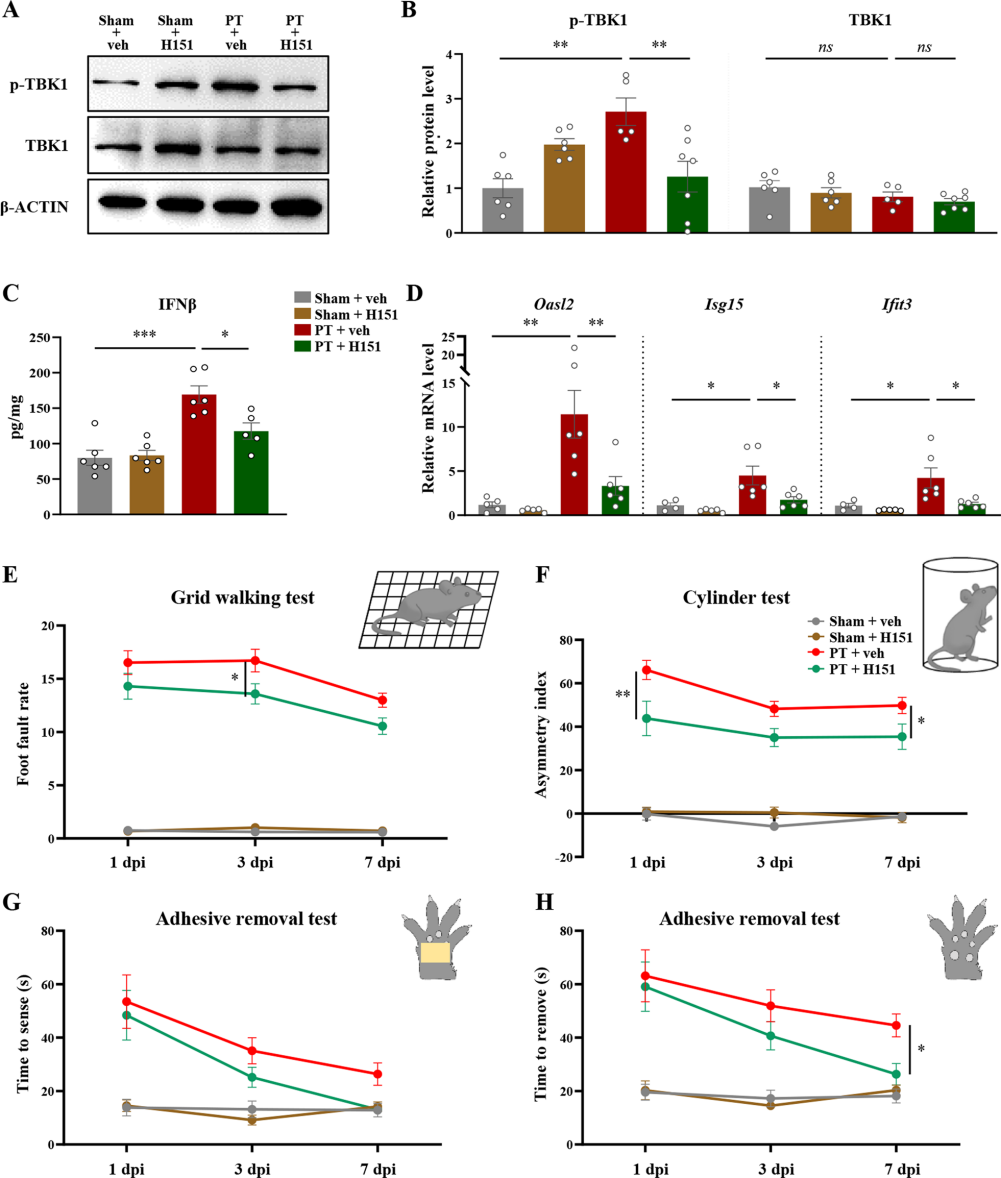
H151 inhibited STING activation and promoted post-stroke recovery of motor function. **A** Representative bands of phospho- and total-TBK1, as well as β-ACTIN using Western Blotting experiments. **B** The relative expression levels of phospho- and total-TBK1. β-ACTIN was used as the internal reference. The results were relative to sham + vehicle group. **C** The protein levels of IFNβ detected by ELISA. **D** The relative mRNA levels of interferon-stimulated genes (*Oasl2*, *Isg15*, and *Ifit3*). The results were relative to sham + vehicle group. n = 4–7 mice per condition. Each dot represented an individual mouse. **E** The quantification of foot fault rate in grid walking test at different time points after photothrombotic stroke. **F** The quantification of forelimb asymmetry in cylinder test. **G** The time spent by the injured forelimb to sense the adhesive tape in adhesive removal test. **H** The time to remove tape in adhesive removal test. n = 12–13 mice for behaviour assessment. Data were presented as mean ± SEM. *P < 0.05, **P < 0.01, ***P < 0.001, ns: not significant

H151 is a highly potent and selective small-molecule covalent antagonist of STING [36]. H151 was intraperitoneally administrated for 8 consecutive days, commencing 1 h after photothrombotic stroke. 7 days after injury, increased levels of phosphorylated TBK1 was observed, which indicated STING signaling activation [35]. H151 treatment could reduce TBK1 phosphorylation, while the total levels of TBK1 remained unchanged (F(3, 20) = 7.538, *P* = 0.0015, Fig. 2A and B). Similarly, IFNβ expression level was also suppressed by H151 (F(3, 19) = 16.10, *P* < 0.0001, Fig. 2C), accompanied by a reduction in the mRNA levels of interferon-stimulated genes (*Oasl2, Isg15, Ifit3*) after stroke (*Oasl2*: F(3, 18) = 10.13, *P* = 0.0004; *Isg15*: F(3, 17) = 7.613,* P* = 0.0019; *Ifit3*: F(3, 17) = 6.534, *P* = 0.0039, Fig. 2D). The STING protein levels were also significantly reduced by H151 treatment (F(3, 16) = 81.32, *P* < 0.0001, Additional file 3: Fig. S3A, B). These results showed that STING signaling activation could be potently inhibited by H151 administration. We then investigated the effects of H151 on stroke-induced motor function deficits. Grid walking test (Fig. 2E) and cylinder test (Fig. 2F) were used to evaluate the forelimb motor impairments and forelimb-use asymmetries, respectively [37, 38]. After PT injury, mice showed obvious deficits in these behavioral tests, while H151 treatment could improve their performance in grid walking test at 3 dpi (group main effect, F(3, 135) = 348.9,* P* < 0.0001, Fig. 2E) and in cylinder test (group main effect, F(3, 128) = 159.1, *P* < 0.0001, Fig. 2F) at 1 and 7 dpi. We further conducted adhesive removal test for assessing sensory-motor impairments (Fig. 2G, H). Injured forelimb tended to spend longer time to sense and remove the adhesive tape after stroke, while H151 treatment could significantly shorten the removal time (group main effect, F(3, 135) = 32.93, *P* < 0.0001, Fig. 2H). Altogether, these results showed that the inhibition of STING signaling activation by H151 could significantly enhance the post-stroke recovery of mice.

### STING inhibition by H151 could suppress the overactivation of microglia after stroke

**Fig. 3:**
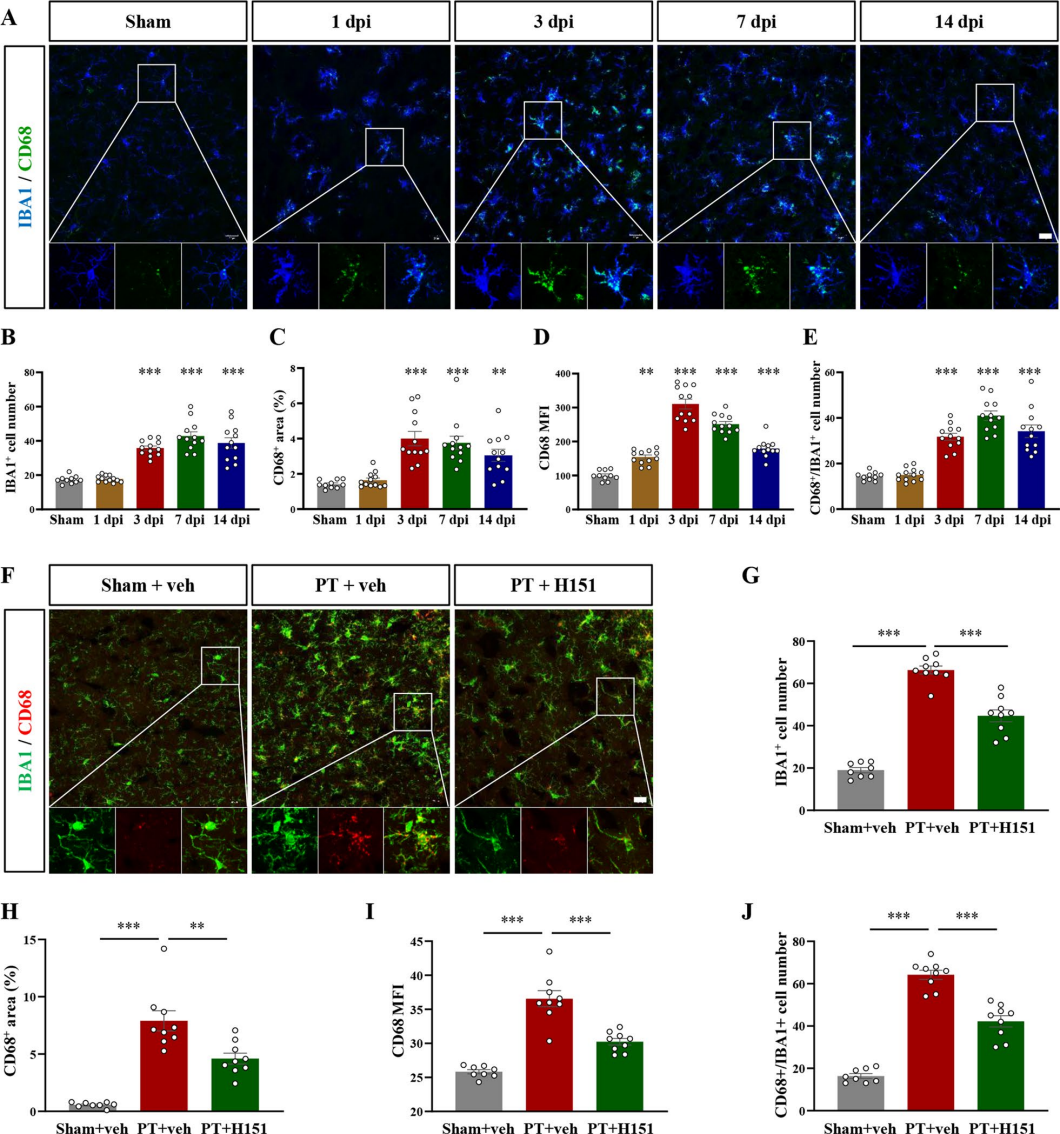
H151 administration reduced CD68 expression in microglia after stroke. **A** Representative immunofluorescent micrographs of IBA1 and CD68 after sham treatment or at different time points after stroke injury. **B** The number of IBA1-positive cells in each FOV in sham or PT group. **C** The percentage of CD68 positive area in each FOV. The results were relative to sham group. **D** The MFI of CD68 after sham or injury. **E** The number of CD68- and IBA1-double positive cells after sham or injury. n = 10–12 FOV from 3 mice. **F** Representative images of IBA1 and CD68 after administration of H151 or vehicle in sham-treated or PT-injured mice. Boxed regions were enlarged for analyzing colocalization. **G**–**J** The number of IBA1-positive cells (**G**), the percentage of CD68 positive area (**H**), CD68 MFI (**I**), and CD68/IBA1-double positive cells were quantified after H151 or vehicle administration at 7 days after sham or stroke. n = 8–9 FOV from 3 mice. Scale bar = 20 μm. Data were presented as mean ± SEM. **P < 0.01, ***P < 0.001

We next investigated whether H151 affected microglia activation by immunostaining IBA1 and CD68 (Fig. 3A), which is considered as a marker for microglia pro-inflammatory activation [39–42]. From 3 dpi after PT modeling, the number of IBA1-positive microglia significantly increased (F(4, 53) = 37.42, *P* < 0.0001, Fig. 3B), accompanied by an increased percentage of CD68-positive area (F(4, 53) = 14.59, *P* < 0.0001, Fig. 3C). The average fluorescence intensity of CD68 began to up-regulate from day 1 after PT modeling (F(4, 53) = 80.61, *P* < 0.0001, Fig. 3D). The number of CD68-positive microglia also upregulated from 3 dpi (F(4, 53) = 43.71, *P* < 0.0001, Fig. 3E), indicating that the microglia around the infarcted cortex was activated after stroke from 3 dpi and remained activated during the subacute phase of stroke. However, after consecutive H151 administration, microglia number was significantly decreased (F(2, 23) = 116.9, *P* < 0.0001, Fig. 3F, G), and CD68 expression level was also reduced in microglia (F(2, 23) = 36.30, *P* < 0.0001, Fig. 3H; F(2, 23) = 47.48, *P* < 0.0001, Fig. 3I; F(2, 23) = 122.4, *P* < 0.0001, Fig. 3J). This indicated that H151 administration could inhibit microglia activation.

**Fig. 4:**
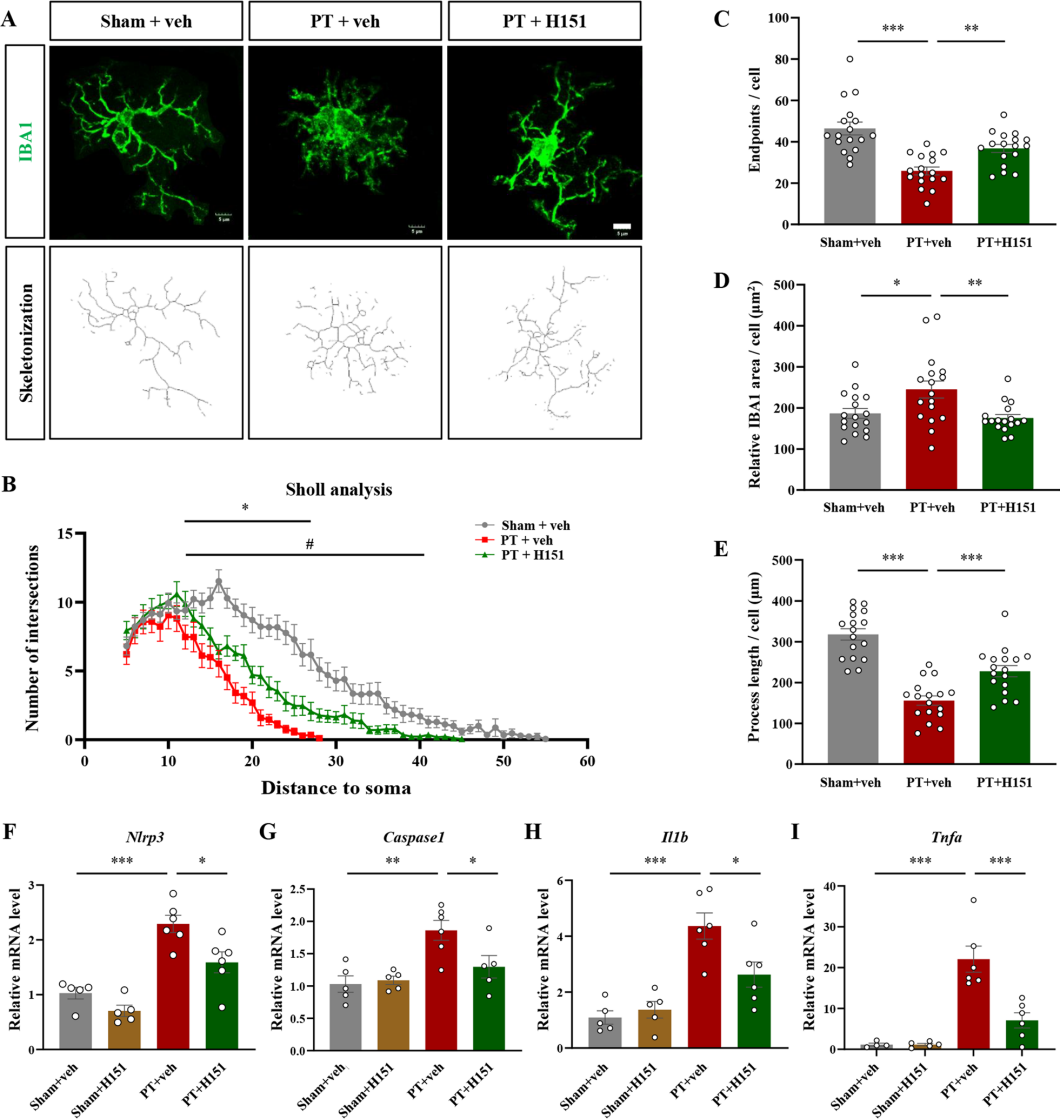
STING inhibition affected the morphological features of microglia and reduced the levels of inflammatory cytokines. **A** Representative skeletonization of IBA1-labelled microglia. Scale bar = 5 μm. **B**–**E** Sholl analysis at 7 days after surgery for intersection numbers (**B**), endpoints (**C**), IBA1-positive area (**D**), and mean process length (**E**) per cell after consecutive administration of vehicle or H151. n = 17 cells from 3 mice per condition. Each dot represented an analyzed cell. **F**–**I** mRNA levels of *Nlrp3* (**F**), *Caspase1* (**G**), *Il1β* (**H**), and *Tnfα* (**I**) were detected by qRT-PCR at 7 days after surgery. n = 4–6 mice per condition. Each dot represented an individual mouse. Data were presented as mean ± SEM. *P < 0.05, **P < 0.01, ***P < 0.001

We further analyzed the morphological changes of IBA1-positive microglia after H151 treatment (Fig. 4A). 7 days after stroke, microglia showed typical activated morphology with a reduced number of cellular processes (F(2, 2439) = 324.5, *P* < 0.0001, Fig. 4B; F(2, 48) = 18.30, *P* < 0.0001, Fig. 4C), enlarged cell body (Fig. 4D), and decreased process length (F(2, 48) = 38.28, *P* < 0.0001, Fig. 4E), while after H151 treatment microglia exhibited a more ramified morphology (Fig. 4A–E). We also evaluated the effects of H151 on inflammatory molecules after stroke and found that STING inhibition could significantly reduce the mRNA levels of *Nlrp3* (F(3, 18) = 21.20, *P* < 0.0001, Fig. 4F)*, Caspase 1* (F(3, 17) = 7.969, *P* = 0.0016, Fig. 4G)*, Il1β* (F(3, 18) = 14.32, *P* < 0.0001, Fig. 4H)*,* and *Tnfα* (F(3, 17) = 21.93, *P* < 0.0001, Fig. 4I). Altogether, these results showed that STING inhibition by H151 could reduce the number of CD68-positive microglia, restore microglia morphology to a more ramified state, and suppress neuroinflammation after stroke.

### Microglia-mediated synapse phagocytosis was inhibited after STING inhibition

**Fig. 5:**
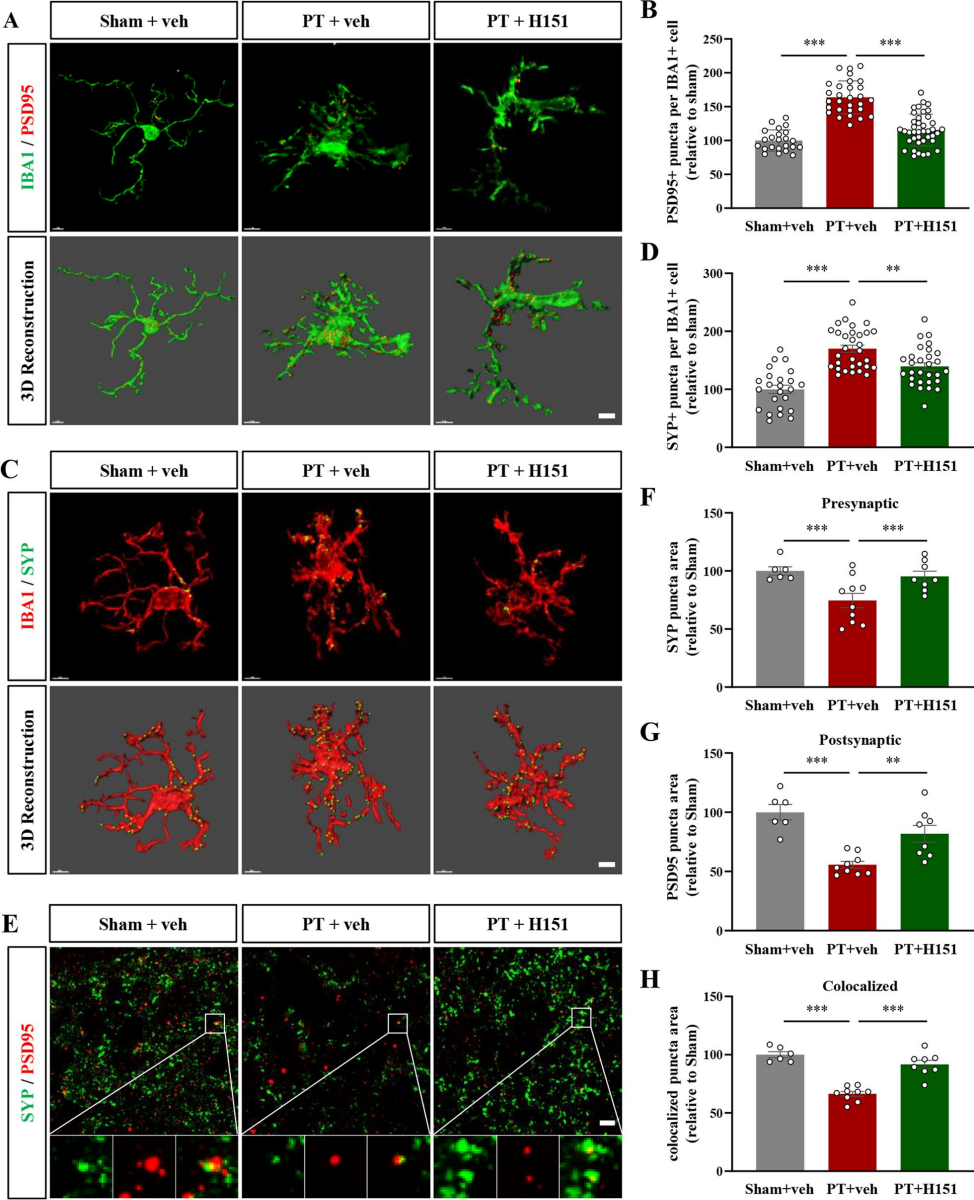
STING inhibition by H151 suppressed microglial phagocytosis of synapses after stroke. **A** Representative micrographs and 3D reconstructions of IBA1 and PSD95 at 7 days after surgery. **B** Quantitative data of PSD95 puncta number in IBA1-positive microglia. **C** Representative micrographs and 3D reconstructions of IBA1 and SYP after vehicle or H151 treatment. **D** Quantification of SYP puncta number in IBA1-positive microglia. **E** Representative images of SYP and PSD95. Boxed regions were enlarged for analyzing colocalization. **F** Relative SYP puncta number. **G** Relative puncta number of PSD95. **H** The quantification of SYP/PSD95 colocalized puncta number. The results were relative to sham + vehicle group. In (**A**–**D**), n = 23–40 cells from 3 mice per condition. Each dot represented an analyzed cell. In (**E**–**H**), n = 6–10 FOV from 3 mice per condition. Each dot represented a FOV. Scale bar = 5 μm. Data were presented as mean ± SEM. **P < 0.01, ***P < 0.001

**Fig. 6:**
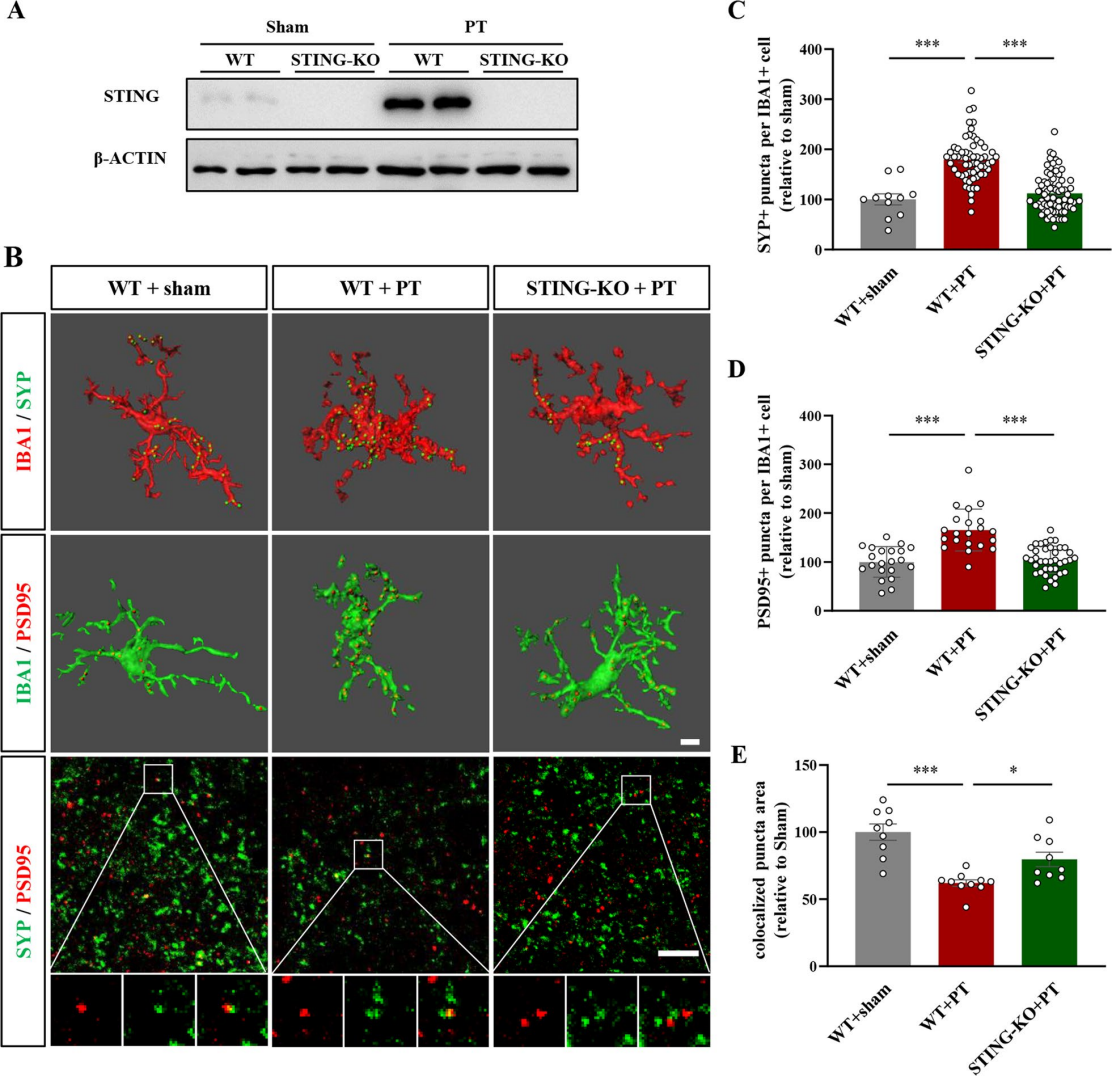
Genetically deleting STING inhibited microglial phagocytosis of synaptic elements after stroke. **A** The protein expression levels of STING in wildtype mice and STING-knockout mice. **B** Representative 3D reconstructions of IBA1 and synaptic elements, along with representative images of SYP and PSD95 at 7 days after surgery. **C** and **D** The quantification of SYP puncta number (**C**) and PSD95 puncta number (**D**) in IBA1-positive microglia. **E** The quantification of SYP/PSD95 colocalized puncta number. The results were relative to sham-treated wildtype mice. In (**C**, **D**), n = 11–74 cells from 3 mice per condition. Each dot represented an analyzed cell. In (**E**), n = 9–10 FOV from 3 mice per condition. Each dot represented a FOV. Scale bar = 5 μm. Data were presented as mean ± SEM. *P < 0.05, ***P < 0.001

Previous studies have shown that microglia are involved in synapse elimination during both the acute and subacute phases after stroke [5, 6]. Similarly, we also found obvious colocalization of IBA1 and post-synaptic marker PSD95 (Fig. 5A) or pre-synaptic marker SYP (Fig. 5C) at 7 days after PT injury. However, after consecutive H151 treatment, there was a significant reduction in synapse engulfment by microglia (F(2, 89) = 62.62, *P* < 0.0001, Fig. 5B; F(2, 81) = 28.49, *P* < 0.0001, Fig. 5D). We further conducted immunofluorescence triple staining for IBA1, PSD95, and CD68 to show that the synaptic materials might be digested by lysosomes of microglia (Additional file 2: Fig. S2A). Similarly, increased colocalization of IBA1, PSD95, and CD68 was observed after stroke injury, which could be significantly decreased after STING inhibition (F(2, 160) = 75.45, *P* < 0.0001, Additional file 2: Fig. S2B). Consist with previous studies [6], we also found obvious synaptic loss around the infarcted cortex (Fig. 5E). However, there was more puncta numbers of SYP (F(2, 21) = 6.589, *P* = 0.0060, Fig. 5F), PSD95 (F(2, 20) = 15.51, *P* < 0.0001, Fig. 5G), and their colocalization (F(2, 20) = 39.72, *P* < 0.0001, Fig. 5H) in PT + H151 group, indicating much synapses were preserved after STING inhibition. We further verified these using STING knockout mice (STING-KO mice, Fig. 6A). Similar to H151 treatment, the microglia in STING-KO mice engulfed less presynaptic and post-synaptic elements (SYP: F(2, 141) = 48.38, *P* < 0.0001, Fig. 6C; PSD95: F(2, 77) = 27.44, *P* < 0.0001, Fig. 6D) after stroke, accompanied by increased synaptic density around the infarcted region (F(2, 25) = 16.31,* P* < 0.0001, Fig. 6B, E). Together, these experiments showed that STING inhibition could suppress the phagocytotic ability of microglia against stroke-affected synapses and promote the recovery of synaptic density.

### STING inhibition down-regulated several phagocytosis-related molecules

**Fig. 7:**
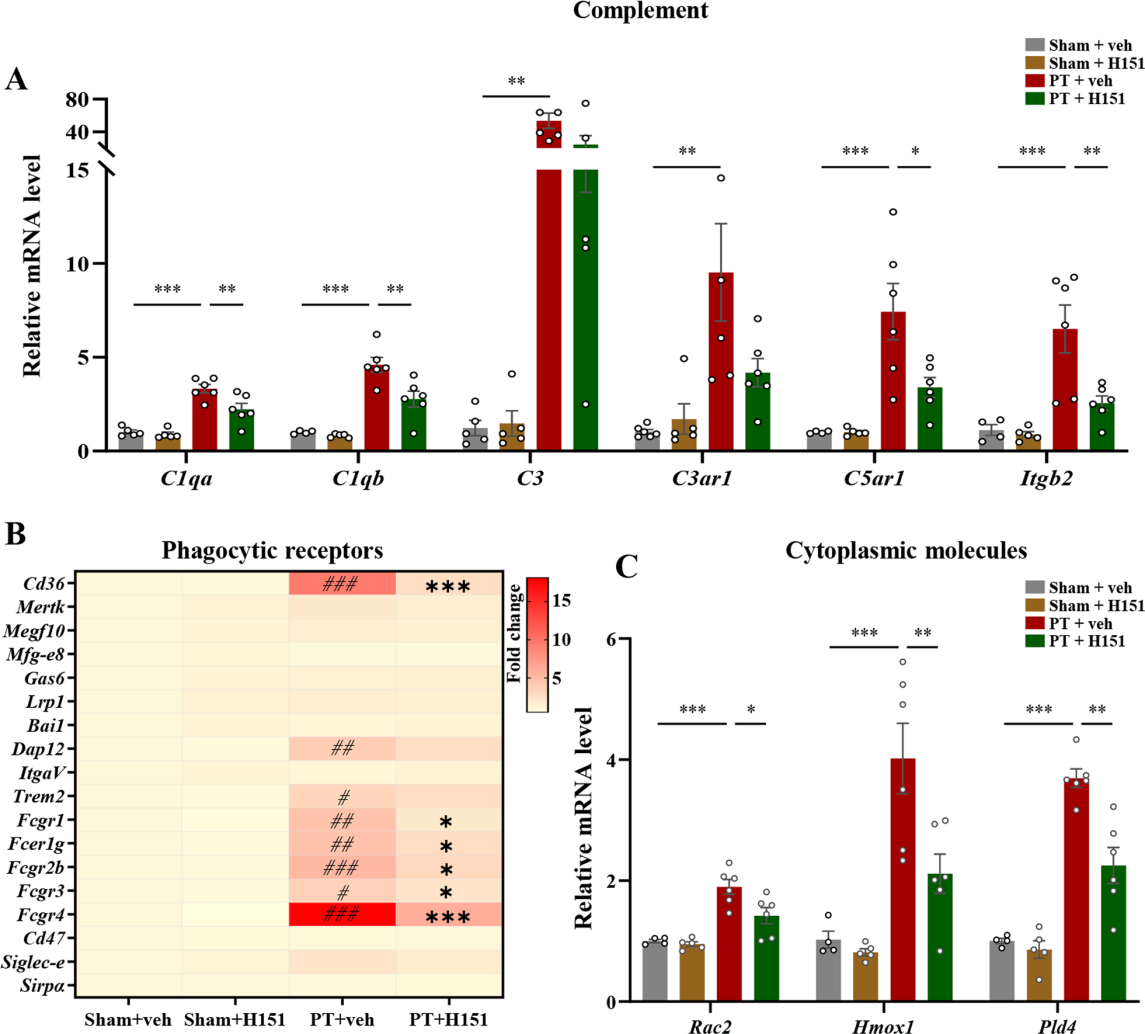
Antagonizing STING activation decreased the levels of phagocytosis-related molecules. **A** The relative mRNA levels of complete components after vehicle or H151 treatment at 7 days after surgery. **B** Heat map of the relative mRNA levels of phagocytic receptors. Darker color represented higher fold change. ^#^*P* < 0.05, ^##^*P* < 0.01, ^###^*P* < 0.001, compared with sham + vehicle group; *P < 0.05, ***P < 0.001, compared with PT + vehicle group. **C** The fold change of mRNA levels of *Rac2*, *Hmox1*, and *Pld4* after vehicle or H151 treatment. The results were relative to sham + vehicle group. n = 4–6 mice per condition. Data were presented as mean ± SEM. *P < 0.05, **P < 0.01, ***P < 0.001

We moved on to explore how STING was involved in microglial phagocytosis. Complement family had been proven to mediate the phagocytic recognition of targeted synapses [43], we therefore analyzed the mRNA levels of complement components. 7 days after PT injury, *C1qa*, *C1qb*, *C3*, *C3ar1*, *C5ar1*, and *Itgb2* was upregulated relative to sham-treated group (Fig. 7A). Among these targets, *C1qa*, *C1qb*, *C5ar1*, and *Itgb2* could be significantly decreased after STING inhibition. H151 administration did not affect the levels of these molecules in sham-treated mice (*C1qa*: F(3, 18) = 26.32, *P* < 0.0001; *C1qb*: F(3, 17) = 27.71, *P* < 0.0001; *C3*: F(3, 18) = 9.906, *P* = 0.0004; *C3ar1*: F(3, 19) = 7.103, *P* = 0.0022; *C5ar1*: F(3, 17) = 11.06, *P* = 0.0003; *Itgb2*: F(3, 17) = 11.60, *P* = 0.0002, Fig. 7A). In addition to complement components, we also measured the levels of the known phagocytic receptors expressed in microglia [44, 45]. Of the detected receptors, *Cd36* and Fc family receptors (*Fcgr1*, *Fcer1g*, *Fcgr2b*, *Fcgr3*, and *Fcgr4*) were sensitive to STING inhibition (*Cd36*: F(3, 18) = 4.878, *P* = 0.0118; *Dap12*: F(3, 18) = 13.31, *P* < 0.0001; *Trem2*: F(3, 18) = 13.15, *P* < 0.0001; *Fcgr1*: F(3, 17) = 14.37, *P* < 0.0001; *Fcer1g*: F(3, 17) = 15.53, *P* < 0.0001; *Fcgr2b*: F(3, 17) = 20.69, *P* < 0.0001; *Fcgr3*: F(3, 17) = 28.46, *P* < 0.0001; *Fcgr4*: F(3, 17) = 29.87, *P* < 0.0001, Fig. 7B). The process of phagocytosis also requires the conduction of intracellular signaling molecules [46–48]. We therefore detected the intracellular phagocytosis-related signaling molecules, and found that the transcriptional levels of *Rac2*, *Pld4*, and *Hmox1* could be significantly down-regulated after STING inhibition (*Rac2*: F(3, 17) = 17.30, *P* < 0.0001; *Hmox1*: F(3, 17) = 14.45, *P* < 0.0001; *Pld4*: F(3, 17) = 41.74, *P* < 0.0001, Fig. 7C). These results showed that many phagocytosis-related molecules were under the regulation of STING signaling.

**Fig. 8:**
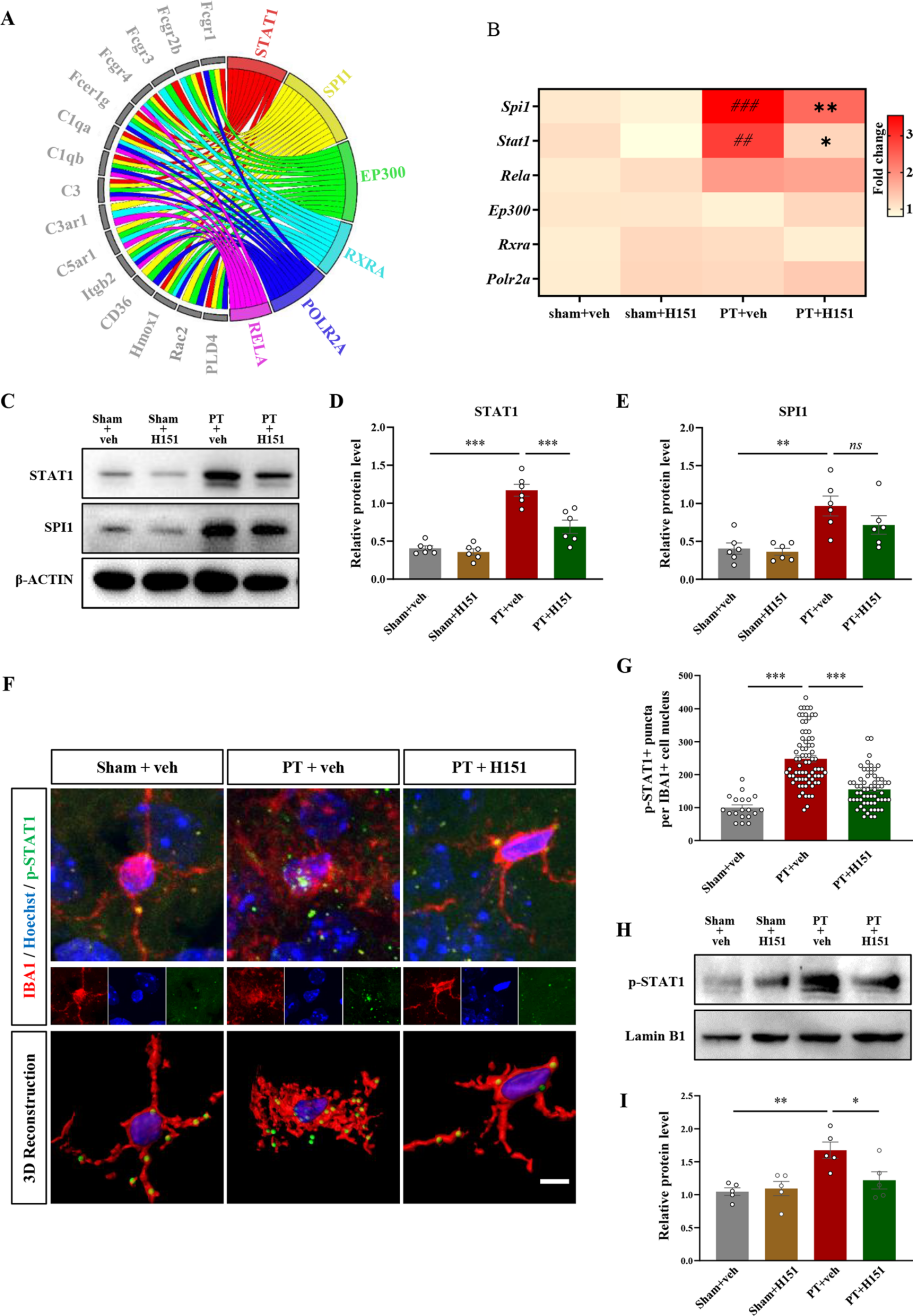
H151 inhibited STAT1 expression and nucleus translocation. **A** Chordal graph showing the regulatory relationship between transcriptional factors and phagocytosis-related molecules based on the database of Cistrome Data Browser. **B** Heat map of the mRNA levels of the transcriptional factors that potentially regulated phagocytosis. Darker color represented higher fold change. ^##^*P* < 0.01, ^###^*P* < 0.001, compared with sham + vehicle group; *P < 0.05, compared with PT + vehicle group. n = 4–6 mice per condition. **C** Representative bands of STAT1 and SPI1 in each experimental condition. **D**, **E** Quantitive analysis of protein expression levels STAT1 (**D**) and SPI1 (**E**). n = 6 mice per condition. **F** Representative immunofluorescent micrographs and 3D reconstructions of phosphorylated STAT1, IBA1, and Hoechst. Scale bar = 5 μm. **G** The relative p-STAT1 puncta number in the nucleus of IBA1-positive cell. n = 19–74 cells from 3 mice. Each dot represented an analyzed cell. **H** Representative bands of phospho-STAT1 and Lamin B1. The nuclear fractions were used for Western Blotting. **I** Quantitive analysis of protein expression levels of phospho-STAT1. Lamin B1 was used as internal control for nuclear protein. Data were presented as mean ± SEM. *P < 0.05, **P < 0.01, ***P < 0.001, ns: not significant

We next asked how STING achieved this regulation. We hypothesized that these phagocytosis-related molecules might be regulated by one or more shared transcriptional factors, and some of the transcriptional factors were the downstream effectors of STING signaling. Using the database of Cistrome Data Browser, several candidate transcriptional factors were predicted to regulate the majority of the above-mentioned molecules (Fig. 8A). After further verification by qRT-PCR and Western Blotting, STAT1 was chosen for further investigation because it was the only transcriptional factor that could be down-regulated in both mRNA (*Spi1*: F(3, 17) = 26.25, *P* < 0.0001; *Stat1*: F(3, 17) = 8.481, *P* = 0.0012, Fig. 8B) and protein levels (STAT1: F(3, 20) = 33.87, *P* < 0.0001, Fig. 8C, D; SPI1: F(3, 20) = 8.047, *P* = 0.0010, Fig. 8E) after STING inhibition. It is known that the nucleus translocation of phosphorylated STAT1 is required to evoke STAT1-dependent gene expression [49]. For assessing the nuclear distribution of phosphorylated STAT1, we first performed immunostaining against IBA1, phospho-STAT1, and Hoechst. We found that stroke induced obvious nuclear translocation of phospho-STAT1 in microglia (Fig. 8F), which could be significantly inhibited by H151 treatment (F(2, 150) = 50.17, *P* < 0.0001, Fig. 8G). For more accurate quantification, we extracted the nuclear fraction and used for Western Blotting. Low levels of GAPDH were detected in the nuclear extracts, indicating little contamination from cytoplasmic fractions under our experimental conditions (Additional file 4: Fig. S4A). We found increased protein levels of phospho-STAT1 in the nuclear fraction at 7 days after stroke, and H151 administration reduced the nuclear distribution of phospho-STAT1 (F(3, 16) = 6.907, *P* = 0.0034, Fig. 8H, I). Altogether, these results indicated that STING intimately regulated various phagocytosis-related molecules and their transcriptional factor STAT1.

## Discussion

In this study, we used a well-characterized STING inhibitor H151 to reveal the regulatory role of STING in microglia-mediated synapse engulfment after stroke injury. We showed that STING affected various phagocytosis-related molecules probably through regulating STAT1 phosphorylation and nucleus translocation. Most previous studies primarily focused on the intimate relationship between STING activation and inflammatory cytokines induction. To our knowledge, this is the first study reporting STING is closely involved in microglial phagocytosis of synapses after stroke.

Except for regulating neuroinflammation, the phagocytotic ability of microglia has recently been considered as another important determinant for post-stroke recovery [50]. Immediately after focal brain ischemia, microglia migrate to the infarct region and engulf dead/dying cells and cell debris, this clearance process is generally considered beneficial for resolving neuroinflammation, promoting remyelination, and facilitating network reorganization [51, 52]. However, activated microglia might also excessively engulf stressed-but-viable neurons and synapses in the ischemic penumbra during disease progression, thereby exacerbating brain injury [53]. These studies indicated that microglial phagocytosis after stroke is a complicated biological progress, and the phagocytic activity against different substrates might have varied effects on disease progression. In our study, we mainly discussed the relationship between STING and phagocytosis of synapses. Whether or not STING was also involved in regulating the engulfment of other targets, such as live neurons or myelin debris, at different stages of stroke remains to be further discussed in the future.

Previous studies have implied the potential association between STING and cell phagocytosis. By conducting the global proteomic analyses between STING-harboring and STING-knockout macrophages, researchers have found that several differentially expressed proteins were related to phagocytosis [54]. It was also reported that LPS could induce the engulfment of fluorescent latex beads by BV2 cells, and STING agonist 2’3-cGAMP could further enhance the phagocytic ability in vitro. It is noteworthy that cGAMP treatment alone, without LPS induction, failed to promote the phagocytosis of beads [55]. For acute myeloid leukemia, increasing cell turnover rate accelerated the release of mtDNA, which was processed by bone marrow-derived macrophages via LC3-associated phagocytosis and activated cGAS/STING signaling. STING inhibition by H151 in macrophages could decrease the phagocytic potential and increase tumor progression [56]. These studies provided several hints for the regulatory roles of STING in phagocytosis. In our study, we further verified that STING activation has a significant influence on complement components, Fc family receptors, and other phagocytosis-related molecules, thereby affecting microglial phagocytosis of synapses after stroke. Whether or not STING mediates similar effects in other kinds of immune cells and under other pathological conditions remains an open question.

It is known that pruning unnecessary synapses by microglia is critical for normal brain development [57, 58]. However, following pathological stimuli, excessive phagocytosis of synapses might occur and contribute to cognitive function decline and disease progression [59]. After brain ischemia, accompanied by extensive neuronal death, the synaptic connections to the injured neurons are lost and the original neural network is disrupted. It is now known that some of the synapses in the penumbra are eliminated by microglia and can be salvageable [53]. Microglia contribute to synapse loss through phagocytosis [5, 6, 60] or BDNF signaling [61] after stroke. In our study, we found that after STING inhibition, microglia engulfed decreased numbers of both pre- or post-synaptic marker proteins. We therefore speculated that STING inhibition could rescue stressed synapses and alleviate neural circuit disruption, which might be beneficial for attenuating the burden of network remodeling and promoting post-stroke recovery [62]. On the other hand, we also found that several pro-inflammatory cytokines were downregulated after H151 treatment, which is consistent with previous findings using another STING inhibitor C176 [19, 22] and STING knockout mice [24]. These studies also reported enhanced recovery after STING inhibition. Together, we believed that STING might intimately regulate microglia activity, and be closely associated with two major functions of microglia: inflammatory cytokines release and phagocytosis. After STING was inhibited, over-activated microglia might be tamed, resulting in less neuroinflammation and more preserved synapses, thereby contributing to post-stroke recovery.

## Conclusion

Our study showed that antagonizing STING by H151 after stroke could inhibit microglia activation and decrease their phagocytic ability of synapses around the infarcted region, which alleviates the stroke-induced motor functional deficits. STING might regulate phagocytosis-related molecules by affecting STAT1 expression and nucleus translocation of phosphorylated STAT1. Our study uncovered an intimate relationship between STING activation and microglia-mediated synapse removal, hoping to provide novel insights into the complex roles of STING and suggest a potential drug-able target for post-stroke recovery.

## Methods

### Animals

All experimental protocols and animal handling procedures were approved by the Animal Research Ethics Committee of China Pharmaceutical University and carried out according to the guidelines and regulations. All animals were raised under pathogen-free conditions in the Animal Centre of China Pharmaceutical University and kept on a standard 12-h light-to-dark cycle with ad libitum access to food and water. 6- to 8-week-old male C57BL/6 mice were purchased from Vital River Laboratory Animal Technology, China. STING knockout mice were purchased from GemPharmatech, China.

### Animal surgery and drug administration

Mice were intraperitoneally administered with Rose Bengal solution (Sigma, USA; 10 mg/ml in saline, 100 mg/kg of body weight) or vehicle. A cold light source (2 mm diameter, 30,000 lx) was placed gently to the exposed skull (2 mm lateral from Bregma) for 15 min to induce photothrombosis. For STING inhibition, H151 (MedChem Express, USA) was first intraperitoneally administered (750 nmol for each mouse) one hour after photothrombotic stroke and every 24 h for 7 days. H151 was stored in – 20 ℃ and dissolved in 5% DMSO and 10% Tween 80.

### Western blotting

7 days after surgery, the sensorimotor cortex adjacent to the infarcted core was dissected, and protein was extracted using RIPA buffer with PMSF (Beyotime, China). The protein levels were measured using the BCA protein assay kit (Vazyme, China) according to the manufacturer’s instructions. 30–50 μg protein per lane was loaded on SDS polyacrylamide gel and transferred to polyvinyl-difluoride membranes (Millipore, USA). The membranes were blocked using 5% non-fat milk or bovine serum albumin in Tris-buffered saline containing Tween 20 (TBST). Then, the membranes were incubated overnight at 4 ℃ with primary antibodies as follows: rabbit anti-STING (1:1000, Proteintech, 48,853), rabbit anti-TBK1 (1:1000, Cell Signaling Technology, 3504), rabbit anti-PTBK1 (1:1000, Abcam, ab109272), rabbit anti-cGAS (1:1000, Cell Signaling Technology, 31,659), mouse anti-GAPDH (1:10,000, Proteintech, 60,004–1-Ig), rabbit anti-Phospho-Stat1 (1:1000, Cell Signaling Technology, 9167), and rabbit anti-β-ACTIN (1:10,000, ABclonal, AC050). After incubation, the membranes were washed three times with TBST, and further incubated with HRP-conjugated goat anti-rabbit/mouse IgG secondary antibodies for 1 h at room temperature. After washing three times, the bands were detected using the enhanced chemiluminescence (ECL) advance kit (Vazyme, China) and quantified using ImageJ software (USA).

### Immunofluorescence

Mice were anesthetized with isoflurane and perfused with 0.9% saline and cold 4% paraformaldehyde (PFA). The brain was removed and immersed in 4% PFA overnight at 4 ℃. 20% and 30% sucrose solution was used for dehydrating the brain. The brain was then cut into 16 μm thick sections using the Leica-1950 cryostat (Leica Instruments, Germany). After rewarming, the sections were washed twice using phosphate buffer saline (PBS) and blocked with 10% goat serum and 0.1% Triton at room temperature for 1 h. Then, the following primary antibodies were used for incubation overnight at 4 ℃: rabbit anti-STING (1:500, Proteintech, 48,853), mouse anti-IBA1 (1:400, Millipore, MABN92), rabbit anti-IBA1 (1:400, Wako, 019–19741), mouse anti-NeuN (1:400, Millipore, MAB377), mouse anti-GFAP (1:400, Millipore, MAB360), rat anti-CD68 (1:400, Abcam, ab53444), rabbit anti-SYP (1:800, Abcam, ab32127), rabbit anti-Phospho-Stat1 (1:500, Cell Signaling Technology, 9167 and 7649), rabbit anti-Lamin B1 (1:5000, Abways, AB0054) and mouse anti-PSD95 (1:400, Invitrogen, MA1-046). The next day, the primary antibodies were abolished and sections were washed with PBS for three times. Then, the sections were incubated in Alexa Flour 488 conjugated goat anti-rabbit IgG (1:500, Invitrogen, USA), Alexa Flour 633 conjugated goat anti-rat IgG (1:500, Invitrogen, USA), or Alexa Flour 633 conjugated goat anti-mouse IgG (1:500, Invitrogen, USA) for 1 h at room temperature. The sections were rinsed and mounted on microscope slides with mounting media (Beyotime, China). The fluorescent images were acquired using the Olympus FV3000 confocal microscope (Olympus, Japan). The mean fluorescence intensity (MFI) and the area of positive signals are analyzed by ImageJ software (USA).

For microglia morphologic analysis, the sections were immunostained with IBA1 and fluorescent secondary antibody. The microglial traces were generated and analyzed using the Sholl analysis plugin in ImageJ.

For synaptic density and elimination analysis, the sections were stained with IBA1, SYP, PSD95, or CD68 depending on the purpose of the experiment. The z-stacks were acquired using a 100 × oil objective with a step size of 0.75 μm. The acquired images were 3D reconstituted by Imaris software (UK). The engulfed synapses in microglia and synaptic puncta were detected and quantified using the colocalization function and spots detection function of Imaris.

### ELISA assays

To probe the levels of IFN-β, the dissected cortex around the infarcted region was lysed using RIPA buffer (Beyotime, China), and the total protein concentration was quantified using BCA protein assay kit (Vazyme, China). IFN-β levels were measured using the mouse IFN-β ELISA kits (AiFang Biological, China) according to the manufacturer’s protocol.

### qRT-PCR

The total RNA of the peri-infarcted cortex was extracted using TRIzol reagent (Vazyme, China), and quantified by NanoDrop 2000 (Thermo Fisher Scientific, USA). RNA was then reversely transcribed by HiScript 1st Strand cDNA Synthesis Kit (Vazyme, China) according to the manufacturer’s protocol. Quantitative PCR was performed using SYBR Green Master Mix (Vazyme, China) on an ABI7000 real-time PCR system (Applied Biosystems, USA). The cycle time values were normalized to *β-Actin*. The sequences of primers used for qRT-PCR were listed in Additional file 5: Table S1.

### Behavioral test

The grid walking test and cylinder test were used for assessing the forelimb motor impairments and forelimb-use asymmetries, respectively [37, 38]. For grid walking test, a 13 × 13 mm square wire grid was used, on which mice were allowed to freely walk and videotaped for 4 min. The total number of steps for the contralateral paw was counted. Each time the contralesional side of the paw slipped through an open grid, a “foot fault” was counted. The total number of steps for the contralateral paw was also counted. Foot fault rates were calculated as (foot faults/total steps*100). For cylinder test, mice were placed in a 500-mL glass beaker and videotaped for 4 min. The times of the contralateral paw, ipsilateral paw, or both paws placement on the wall of the beaker were recorded. The asymmetry index is calculated as (non-impaired forelimb contact−impaired forelimb contact)/(non-impaired forelimb contact + impaired forelimb contact + both forelimb contact). For adhesive removal test, two adhesive tapes were applied to the forepaws, and the time to sense and remove were recorded. The maximum time to sense or remove the tape was limited to 90 s. The sense time and removal time used by the non-impaired forepaw were similar between each group. Pre-training for 5 days was conducted before the induction of photothrombotic stroke.

### Nuclear and cytoplasmic separation

For assessing the protein levels of phosphorylated STAT1, NE-PER Nuclear and Cytoplasmic Extraction Reagents (Thermo scientific) were used according to the manufacturer’s protocol. The purity of nuclear extract was verified by detecting GAPDH protein levels. Lamin B1 was used as internal positive control.

### Statistical analysis

Data were analyzed by GraphPad Prism software, version 9.0 (USA) and presented as mean ± SEM. One-way or two-way ANOVA tests followed by Tukey’s or Bonferroni’s post hoc test were used for multiple comparisons. Values with *P* < 0.05 were considered statistically significant.

### Supplementary Information

**Additional file 1: Figure S1.** Photothrombotic stroke led to the upregulation of cGAS. **A** Representative bands of cGAS and GAPDH at different time points after stroke injury. GAPDH was used as the internal reference. **B** The protein expression levels of cGAS were relative to sham group. n = 4 mice per condition. Data were presented as mean ± SEM. **P < 0.01.

**Additional file 2: Figure S2.** H151 inhibited microglial phagocytosis of synaptic protein. **A** Representative micrographs and 3D reconstructions of IBA1, PSD95, and CD68 under different experimental conditions. Scale bar = 5 μm. **B** Quantitative analysis of PSD95- and CD68-double positive puncta number in microglia. n = 38–72 cells from 3 mice per condition. Each dot represented an analyzed cell. Data were presented as mean ± SEM. **P < 0.01, ***P < 0.001.

**Additional file 3: Figure S3.** H151 treatment could decrease the protein levels of STING after stroke. **A** Representative bands of STING at 7 days after stroke injury. β-ACTIN was used as the internal reference. **B** The relative protein expression levels of STING. n = 5 mice per condition. Data were presented as mean ± SEM. **P < 0.01, ***P < 0.001.

**Additional file 4: Figure S4.** Purity validation of nuclear and cytoplasmic protein separation. **A** Representative bands of LaminB1 and GAPDH. Lamin B1 and GAPDH were used as housekeeping proteins for nuclear and cytoplasmic fractions respectively. Total protein represented protein lysed with RIPA lysis buffer.

**Additional file 5: Table S1.** The sequences of primers used for qRT-PCR.

## Data Availability

The data are available from the corresponding author upon reasonable request.

## Abbreviations

STING: Stimulator of interferon genes
SYP: Synaptophysin
PSD95: Postsynaptic density protein 95
STAT1: Signal transducer and activator of transcription 1
cGAS: Cyclic GMP–AMP synthase
dpi: Days post injury
FOV: Field of view
IFNβ: Interferon β
IBA1: Ionized calcium-binding adapter molecule1
GFAP: Glial fibrillary acidic protein
TBK1: TANK-binding kinase 1
IRF3: Interferon regulatory factor 3
