# Supplementary figures and images for: STING inhibition suppresses microglia-mediated synapses engulfment and alleviates motor functional deficits after stroke
Source: J Neuroinflammation. 2024 Apr 8;21:86. doi: 10.1186/s12974-024-03086-8 (PMC11000342; doi:10.1186/s12974-024-03086-8)

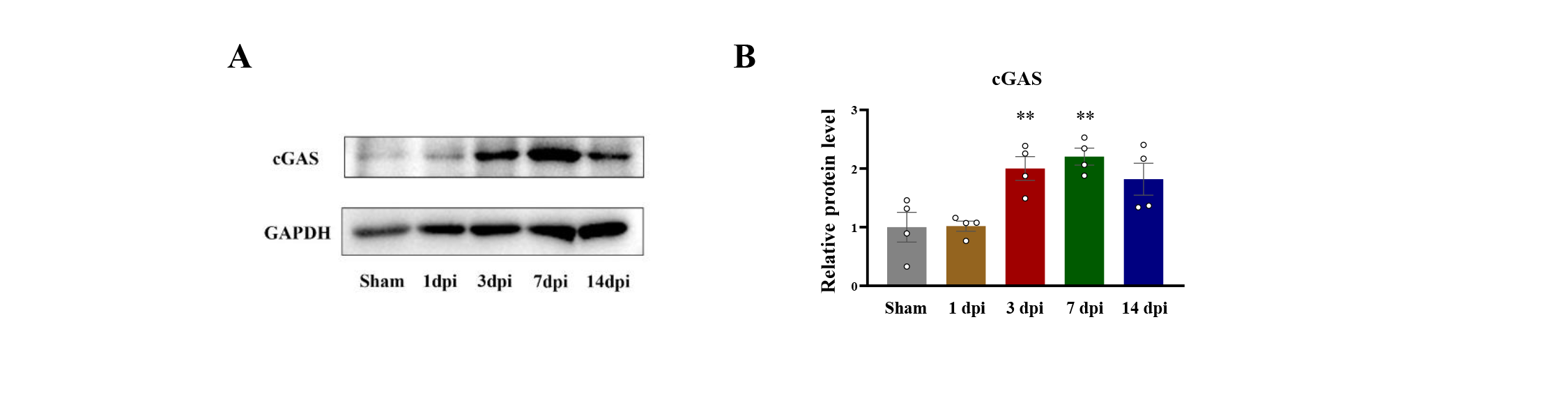

Supplement: Supplementary file 1 — Additional file 1: Figure S1. Photothrombotic stroke led to the upregulation of cGAS. A Representative bands of cGAS and GAPDH at different time points after stroke injury. GAPDH was used as the internal reference. B The protein expression levels of cGAS were relative to sham group. n = 4 mice per condition. Data were presented as mean ± SEM. **P < 0.01. [file 12974_2024_3086_MOESM1_ESM.tif]

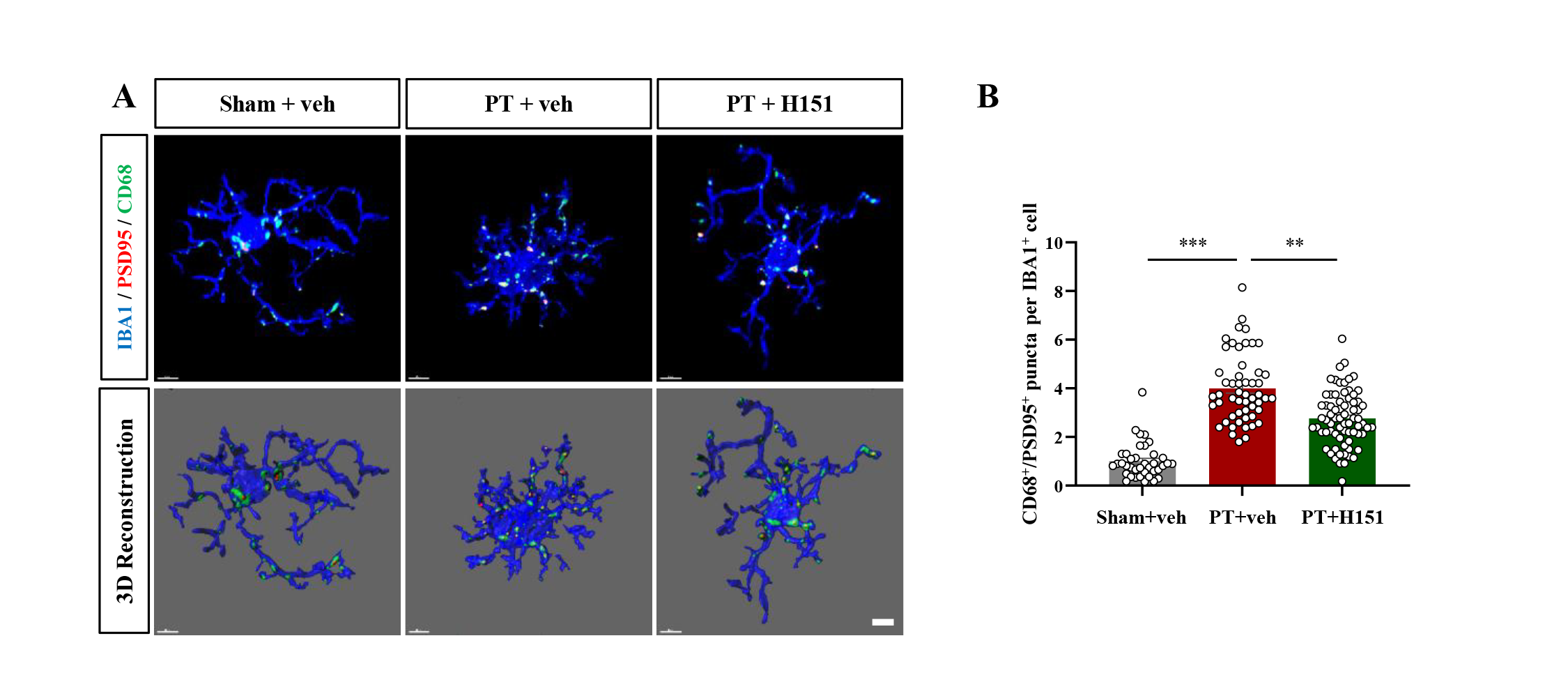

Supplement: Supplementary file 2 — Additional file 2: Figure S2. H151 inhibited microglial phagocytosis of synaptic protein. A Representative micrographs and 3D reconstructions of IBA1, PSD95, and CD68 under different experimental conditions. Scale bar = 5 μm. B Quantitative analysis of PSD95- and CD68-double positive puncta number in microglia. n = 38–72 cells from 3 mice per condition. Each dot represented an analyzed cell. Data were presented as mean ± SEM. **P < 0.01, ***P < 0.001. [file 12974_2024_3086_MOESM2_ESM.tif]

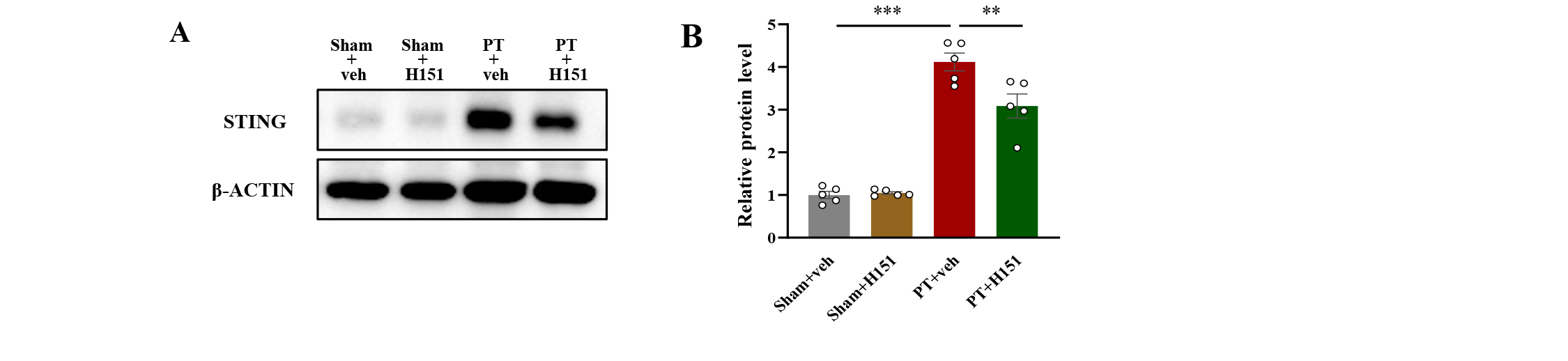

Supplement: Supplementary file 3 — Additional file 3: Figure S3. H151 treatment could decrease the protein levels of STING after stroke. A Representative bands of STING at 7 days after stroke injury. β-ACTIN was used as the internal reference. B The relative protein expression levels of STING. n = 5 mice per condition. Data were presented as mean ± SEM. **P < 0.01, ***P < 0.001. [file 12974_2024_3086_MOESM3_ESM.tif]

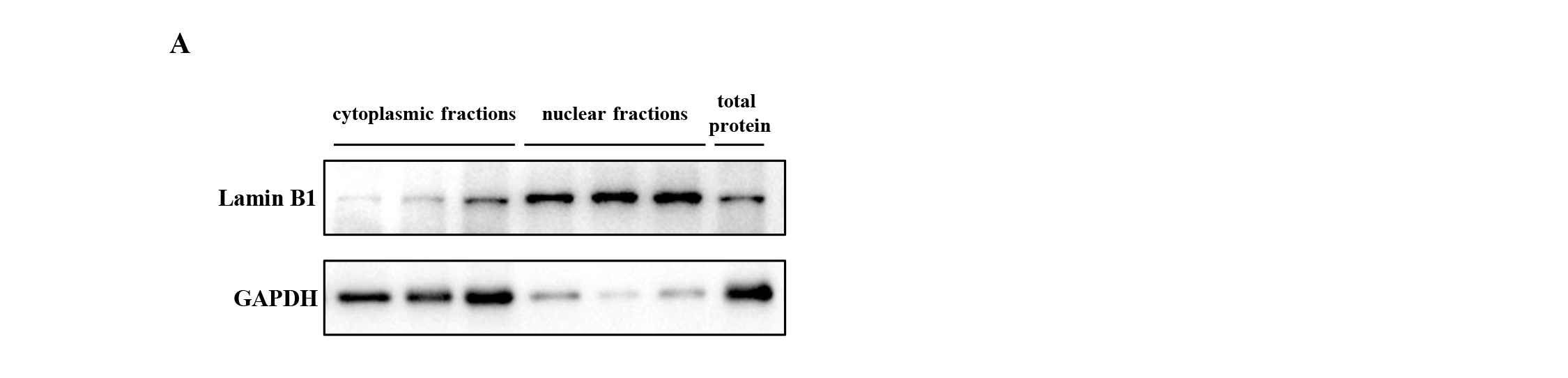

Supplement: Supplementary file 4 — Additional file 4: Figure S4. Purity validation of nuclear and cytoplasmic protein separation. A Representative bands of LaminB1 and GAPDH. Lamin B1 and GAPDH were used as housekeeping proteins for nuclear and cytoplasmic fractions respectively. Total protein represented protein lysed with RIPA lysis buffer. [file 12974_2024_3086_MOESM4_ESM.tif]
