# Supplementary material for: STING inhibition suppresses microglia-mediated synapses engulfment and alleviates motor functional deficits after stroke
Source: J Neuroinflammation. 2024 Apr 8;21:86. doi: 10.1186/s12974-024-03086-8 (PMC11000342; doi:10.1186/s12974-024-03086-8)
Supplement: Supplementary file 5 — Additional file 5: Table S1. The sequences of primers used for qRT-PCR. [file 12974_2024_3086_MOESM5_ESM.doc]

**Supplementary Table 1. The sequences of primers used for qRT-PCR.**

| **Gene name** | **Forward primer (5’-3’)** | **Reverse primer (5’-3’)** |
| --- | --- | --- |
| *Nlrp3* | TCACAACTCGCCCAAGGAGGAA | AAGAGACCACGGCAGAAGCTAG |
| *Caspase1* | GGCACATTTCCAGGACTGACTG | GCAAGACGTGTACGAGTGGTTG |
| *Il1b* | TGGACCTTCCAGGATGAGGACA | GTTCATCTCGGAGCCTGTAGTG |
| *Tnfa* | GGTGCCTATGTCTCAGCCTCTT | GCCATAGAACTGATGAGAGGGAG |
| *Oasl2* | CCAAAACGAGGTCGTCAGGAAC | AGCCACCTGTTCCCATCCCTTT |
| *Isg15* | CATCCTGGTGAGGAACGAAAGG | CTCAGCCAGAACTGGTCTTCGT |
| *Ifit3* | GCTCAGGCTTACGTTGACAAGG | CTTTAGGCGTGTCCATCCTTCC |
| *C1qa* | GTGGCTGAAGATGTCTGCCGAG | TTAAAACCTCGGATACCAGTCCG |
| *C1qb* | CAACCAGGCACTCCAGGGATAA | CCAACTTTGCCTGGAGTCCCAG |
| *C3* | CGCAACGAACAGGTGGAGATCA | CTGGAAGTAGCGATTCTTGGCG |
| *C3ar1* | CTGGCGTAAAGATGAAGACGACC | CCAGTGTCCTTGGAGAATCAGG |
| *C5ar1* | CCATTAGTGCCGACCGTTTCCT | CACGAAGGATGGAATGGTGAGG |
| *Itgb2* | CTTTCCGAGAGCAACATCCAGC | GTTGCTGGAGTCGTCAGACAGT |
| *Cd36* | GGACATTGAGATTCTTTTCCTCTG | GCAAAGGCATTGGCTGGAAGAAC |
| *Mertk* | ATCATCCTCGGCTGCTTCTGTG | ACGACCAGTTGGGAATCCTCCT |
| *Megf10* | CGACAGATCCTGCCAGTGTTAC | CAAAAGGCTCCGTTGTGGCAGT |
| *Mfge8* | GAGCAACAGTGCCAAGGAATGG | ACTGTGGGCTACCTTGTAGGAC |
| *Gas6* | GAACTTGCCAGGCTCCTACTCT | GGAGTTGACACAGGTCTGCTCA |
| *Lrp1* | CGAGAGCCTTTGTGCTGGATGA | CGGATGTCCTTCTCAATGAGGG |
| *Bai1* | CTCCACCATTGATGTCCTGAGG | TCTTCTGCCAGCAGGTTGCTGA |
| *Dap12* | GTGACTTGGTGTTGACTCTGCTG | GATAAGGCGACTCAGTCTCAGC |
| *Itgav* | GTGTGAGGAACTGGTCGCCTAT | CCGTTCTCTGGTCCAACCGATA |
| *Trem2* | CTACCAGTGTCAGAGTCTCCGA | CCTCGAAACTCGATGACTCCTC |
| *Fcgr1* | ACCTGAGTCACAGCGGCATCTA | TGACACGGATGCTCTCAGCACT |
| *Fcer1g* | CAGCTCTGCTATATCCTGGATGC | TCCTGGCTCCGGGTGTTCAGG |
| *Fcgr2b* | CTACTGTGGACAGCCGTGCTAA | TCACCGTGTCTTCCTTGAGCAC |
| *Fcgr3* | TCGGTGTCAAATGGAGCAGACC | CTATGGCACCTTAGCGTGATGG |
| *Fcgr4* | TGACAGTGGCTCCTACTTCTGC | GAGTCCTATCAGCAGGCAGAATG |
| *Cd47* | GGTGGGAAACTACACTTGCGAAG | CTCCTCGTAAGAACAGGCTGATC |
| *Siglece* | GTGTCCACAAGAATGACCATCCG | TGAGCCATTCTTCAGGATTGTGG |
| *Sirpα* | TCATCTGCGAGGTAGCCCACAT | ACTGTTGGGTGACCTTCACGGT |
